# Assessing the adoption of lopinavir/ritonavir oral pellets for HIV‐positive children in Zimbabwe

**DOI:** 10.1002/jia2.25214

**Published:** 2018-12-13

**Authors:** Briony Pasipanodya, Rudo Kuwengwa, Margaret L Prust, Bethany Stewart, Christine Chakanyuka, Tonderayi Murimwa, Jason Brophy, Olawale Salami, Angela Mushavi, Tsitsi Apollo

**Affiliations:** ^1^ Clinton Health Access Initiative – Country Team Harare Zimbabwe; ^2^ AIDS and TB Unit The Ministry of Health and Child Care Harare Zimbabwe; ^3^ Clinton Health Access Initiative, Inc. Boston MA USA; ^4^ The World Health Organization – Country Office Harare Zimbabwe; ^5^ The United Nations Children's Fund – Country Office Harare Zimbabwe; ^6^ Drugs for Neglected Diseases – Africa Regional Office Nairobi Kenya

**Keywords:** paediatric HIV, antiretroviral therapy, Zimbabwe, antiretroviral acceptability, lopinavir/ritonavir

## Abstract

**Introduction:**

Heat‐stable lopinavir/ritonavir (LPV/r) oral pellets were developed to overcome challenges with administration and storage experienced with previously available tablet and syrup forms of LPV/r prescribed to paediatric HIV patients. We report on the adoption of LPV/r pellets for infants living with HIV in the public sector antiretroviral therapy (ART) programme in Zimbabwe.

**Methods:**

Infants aged three months to three years who had been prescribed a LPV/r‐based regimen (including ART‐naïve patients) in fourteen facilities across the country were eligible to receive the pellets. Caregivers were counselled on the new formulation and provided with administration guides. A caregiver questionnaire was administered three to four months after the child initiated on pellets. Data were also extracted from patient ART records.

**Results and discussion:**

One hundred and fifty‐seven children were enrolled (median age: 21 months; interquartile range 11.8 to 29.4). Survey data from 74 caregivers were included for analysis. Eighty‐one per cent of the caregivers preferred pellets while 19% preferred the syrup formulation. Eighty‐nine per cent assessed their child's response to taking the pellets as good or very good. Overall, 46% did not report any challenges while 54% reported one or more challenges with using the pellets. Difficulties with administration included: poor taste (36%; 26 participants); swallowing pellets (16%; 12 participants); finishing the dose (14%; 10 participants); and opening the capsule (10%; seven participants). Caregivers who were not confident to instruct others on pellet administration were 5.64 (95% confidence interval 1.45 to 21.95, *p *=* *0.013) times as likely to experience a challenge.

**Conclusions:**

A large proportion of caregivers preferred pellets to other formulations of LPV/r and reported a good response to pellets; however, they also reported challenges with administration. Counselling should focus on ensuring that caregivers can confidently administer pellets and are able to instruct others, to ensure high uptake and good adherence to treatment. LPV/r pellets may be an acceptable substitute for other available forms of LPV/r for eligible children under three years if they are currently on or in need of LPV/r‐containing regimens; however, challenges with administration still highlight the need for improved drug formulations for paediatric ART patients.

## 1 Introduction

By 2017, 20.9 million of the approximately 36.7 million people living with HIV had access to antiretroviral therapy (ART), representing progress towards the UNAIDS 90‐90‐90 targets to be reached in 2020 1. However, recent improvements in ART coverage have been greater in adults than in children. It was estimated that in 2016 only 43% of children living with HIV were on ART compared to 54% of adults 1. Multiple reasons for this disparity exist including lack of access to well‐formulated and tolerable antiretroviral (ARV) medicines designed to cater for all children across the age spectrum 2, 3.

The 2015 World Health Organization (WHO) guidelines on the use of ARV drugs for treating and preventing HIV infection, recommend either abacavir or zidovudine with lamivudine and lopinavir/ritonavir (LPV/r) as the first‐line ART for all infants and children living with HIV between fourteen days and three years of age 4. Randomized trials have demonstrated the superiority of LPV/r over nevirapine (NVP) in children under three years with respect to both viral suppression and mortality 5. In addition, increasing rates of baseline non‐nucleoside reverse transcriptase inhibitor (NNRTI) resistance, particularly in infants with vertical infection despite maternal treatment with efavirenz‐based therapy, have resulted in a movement to shift first‐line therapies away from NNRTIs and towards LPV/r and integrase inhibitors in the latest WHO guidance 6. Historically, LPV/r has only been available in two formulations for children: as syrup (80 mg/20 mg/mL) and a tablet (100 mg/25 mg per tablet). Children under three often experience challenges swallowing tablets whole (which is required for complete gastrointestinal absorption) and are therefore left with the syrup formulation, which has a very unpalatable taste and must also be kept between 2°C and 8°C until it is dispensed for consumption 7. In low‐resource settings, cold chain systems are limited and cold storage space is often only available for vaccines. In Zimbabwe and elsewhere, this has translated to the low uptake of LPV/r in children, and a large number of children continue to be treated with ART based on clinically inferior NVP syrup.

To address the challenges related to the LPV/r syrup, a new heat‐stable and taste‐masked oral pellet formulation of LPV/r was developed. Available since 2015, the pellets represent an optimized paediatric ARV in Zimbabwe. Prior studies have shown the tolerability of pellets to be similar to that of the syrup 8, although the administration of pellets is however different from the other formulations in that it must follow particular guidelines including that pellets may be mixed with food or liquids, but cannot be chewed, crushed or dissolved 7. Therefore, before rolling out this new formulation on a large scale, information was needed about whether this type of medication could be appropriately administered and is acceptable as a substitute to the LPV/r syrup in Zimbabwe's setting. Specifically, a pilot was conducted to assess the adoption of pellets based on feedback from caregivers of children living with HIV and on ART.

## 2 Methods

A small, initial procurement of pellets was made in Zimbabwe prior to large‐scale rollout of the product, allowing sufficient medications for up to 200 children for a year. In order to ensure that learning from this first procurement could be applied during the later rollout, a pilot was established with the goal of enrolling 200 children living with HIV. Eligible children were enrolled according to the inclusion/exclusion criteria outlined in Table 1. Healthcare workers at 14 health facilities were trained on pellet administration. Eligible children were then prescribed the pellets as part of their treatment regimen. Caregivers were invited to participate in a survey administered twice over the six months to ascertain their experience with administering the medicine and acceptance of the pellets as a potential substitute for other formulations.

**Table 1:** Inclusion/exclusion criteria for patient enrolment

Inclusion criteria	Exclusion criteria
Between three months and thirty‐six months and soon. On ART with LPV/r syrup or soon to be initiated on an LPV/r‐based regimen. Caregiver/infant had not missed more than one follow‐up appointment in accordance with their visit schedule. A primary caregiver provided written consent for his/her child to enrol in the pilot study	Had an active opportunistic infection or co‐morbidities, or otherwise showed clinical symptoms of severe or advanced HIV disease as defined by the WHO clinical staging. Were already on LPV/r tablet formulation without any problems. Were prescribed on medication with a drug–drug interaction with LPV/r

ART, antiretroviral therapy; LPV/r, lopinavir/ritonavir; WHO, World Health Organization.

### 2.1 Data collection

In the period February to July 2017, data were collected using questionnaires administered to caregivers by counsellors and/or nurses at routine visits and to health workers by the study team. Data were also collected from patient records regarding patient immunological status (CD4 percentage), viral load (VL) and weight‐for‐age Z‐score at the time of pellets initiation. For measures that were not available at the time of initiation, latest data from within three months of pellets initiation were used for current patients. For newly initiating patients, VL and CD4 tests were conducted at the time of initiation.

### 2.2 Data analysis

Data from the questionnaires were analysed with STATA 14.2, StataCorp LP (College Station, TX, USA) to assess for trends in administration methods and challenges. Factors associated with challenges were assessed using bivariate logistic regression. Measures of central tendency for immunological test results were also analysed for patient monitoring purposes.

### 2.3 Ethics statement

Approval to undertake the study was granted by Chesapeake institutional review board on 19 April 2017 (Pro00018801). The study also had approval from the Medical Research Council of Zimbabwe and the Medicines Control Authority of Zimbabwe.

## 3 Results and discussion

A total of 157 HIV‐positive children (73 male and 72 female; 12 had missing information on gender) were enrolled and initiated on the pellets. Urban facilities recruited 68% of the patients**.** The median age at the time of initiation on pellets was twenty‐one months (minimum three months, maximum one hundred and ten months; interquartile range 11.8 to 29.4, n = 144). A child above three years (outside inclusion criteria) was allowed to enrol as they could not tolerate any other LPV/r formulation. At the time of pellet initiation, 11.4% (n = 18) of children were ART naïve. Among patients already on treatment, the median duration on ART at baseline was ten months (minimum zero, maximum four years three months). At baseline, 58 patients (40.6%) had a CD4 percentage measurement available (median: CD4% 25.5%) and 53 patients (37.1%) had a baseline VL reading (median: 188 copies/μL). Of 53 children on treatment at baseline with VL result (treated with LPV/r syrup for an average of five months), 19 (36%) had a VL above 1000 copies/mL at baseline while 17 (32%) had an undetectable VL (below 50 copies/mL).

The caregiver experiences questionnaire (Appendix 1) was completed by 74 caregivers who had been administering the pellets for an average of three to four months. The mean age of participating caregivers was 32.0 years (standard deviation (SD) 8.1). Of these, 64 were on ART, with a median duration on ART of 2.1 years (minimum 0, maximum 12.2 years) for the 32 who reported ART start date. 78.6% of the caregivers had a secondary level education or higher.

Over the period of the pilot, a total of 34 clients exited the study, representing a drop‐out rate of 21.6%. These patients had been prescribed the pellets for 2.6 months on average when they exited the study or were determined to be loss to follow‐up. Twenty‐five (73.5%) of the individuals who exited the study were taking the LPV/r solution at baseline and the rest were ART naïve and began treatment taking the oral pellets. Seven per cent of patients exited between weeks 3 and 9 of the study period. The main reasons for the patients withdrawing from the pilot were largely due to the child vomiting and not tolerating the pellets. Other reasons not related to the pellets for exiting are detailed in (Table 2).

**Table 2:** Reported reasons for exiting the LPV/r pellets pilot

Reason for exit	Number of patients (%)	ART naïve	Switched from LPV/r syrup
Vomiting or not tolerating pellets	14 (41.2)	3	11
Lost to follow‐up	7 (20.6)	0	7
Transfer out	5 (14.7)	1	4
Negative confirmatory HIV test^a^	3 (8.8)	1	2
Other reason/unknown^b^	3 (8.8)	0	3
Deceased^c^	2 (5.9)	1	1

ART, antiretroviral therapy; LPV/r, lopinavir/ritonavir.

^a^Children with an initial positive result through rapid testing that was later confirmed as negative through confirmatory DNA PCR testing; ^b^caregiver did not state the reason for exit as they were not obligated to, in line with ethical requirements of the pilot protocol; ^c^the causes of death for the two patients were: pneumonia and pulmonary tuberculosis for each child respectively. These were ruled as unrelated to the LPV/r oral pellets.

Caregivers who completed the survey (n = 74) had been administering the pellets for an average of 3.2 months (SD 1.9 months). Forty‐six per cent (34 participants) reported no challenges with administering the pellets and the remaining 54% (40 participants) reported at least one challenge. The number of times each specific challenge was reported is detailed in (Table 3). The child disliking the taste was the most commonly reported challenge (35.6% of participants), followed by challenges with the child vomiting the medicine (21.6%) and having difficulty swallowing the pellets (16.4%, average age 15 months (SD) 0.92).

**Table 3:** Reported challenges with administering LPV/r oral pellets

Challenge	Caregivers reporting challenge n (%)
Administration challenges	33 (44.6)
Disliking taste	26 (35.6)
Difficulty swallowing	12 (16.4)
Difficulty finishing pellets	10 (13.7)
Difficulty opening capsule	7 (9.6)
Side effects	16 (21.6)
Vomiting	16 (21.6)
Logistical challenges	16 (21.6)
Embarrassed to administer them in public	8 (10.8)
Packaging difficult to carry and move around with	3 (4.1)
Difficulty storing pellets	1 (1.4)
Difficulty with discretely carrying pellets from collection point	1 (1.4)

LPV/r, lopinavir/ritonavir.

The results of the questionnaire section on administration preference showed that 51% of the caregivers administered the pellets in porridge or other soft foods, 31% administered in milk or water and 18% placed the pellets directly in the child's mouth followed on by liquids to wash them down. These practices are shown in relation to the child's feeding method at baseline in (Table 4).

**Table 4:** Caregiver administration methods

Administration method (baseline)	Switched from LPV/r syrup	ART naïve	Exclusively breastfed	Mixed feeding	Solid foods	Total
Directly in infant's mouth	13	0	0	3	10	13
Pellets in milk and/or other liquid	19	3	4	5	13	22
Pellets in porridge or other solid food	32	4	2^a^	7^b^	28	37
Total			6	15	51	72^c^

ART, antiretroviral therapy; LPV/r, lopinavir/ritonavir.

^a^Patients with exclusive breastfeeding and administration in solid food may represent data collection or entry error; ^b^one of the patients in this group had an unknown status on whether they started on oral pellets or were switched at baseline; ^c^two caregivers did not respond to the questions on administration preference.

An analysis was conducted to assess for factors that could be associated with having one or more administration challenges with the pellets. If a caregiver reported that they were not confident to instruct others on administration of the pellets, then they were 5.64 times as likely to report challenges with administration (95% confidence interval (CI) 1.45 to 21.95, *p *=* *0.01). Furthermore, if a caregiver reported that the pellets were not working as well as the syrup, then they were also 7.7 times as likely to report a challenge with administration (95% CI 1.6 to 38.3, *p *=* *0.01).

Although the analysis does not establish any causal relationship between factors, the findings point to several questions that health workers could ask caregivers to screen for administration difficulties. Specifically, health workers could ask “How confident do you feel to instruct others on how to administer the pellets?” warranting additional support should the respondent report he/she is not very confident. This screening could be made more effective by timing it around the three‐week mark post pellet initiation, when more patients in this study were likely to abandon use of the drug.

### 3.1 Acceptability of the LPV/r oral pellets

With regard to how well the caregiver felt the pellets were working for their child, 66.7% (48 participants) of caregivers thought that it was better than the LPV/r syrup, 13.9% (10 participants) thought it was the same as the syrup and the remaining 19.4% (14 participants) did not think that the pellets were as good as the syrup. Furthermore, 53.5% (38 participants) reported that the child's response to the pellets was very good, while only 10.3% (eight participants) reported a poor or very poor response to the pellets.

### 3.2 Clinical outcomes of children initiating treatment with LPV/r oral pellets

Of the 53 patients with a recorded baseline VL test result, 14 had a second VL result at the end of the study period. Of the 19 patients, 12 with a baseline VL above 1000 copies/mL had a repeat VL taken at the end of the study period; eight had achieved viral suppression (median = 156 copies/mL, 38% undetectable) after six months of using the pellets; four still had a VL above 1000 copies/mL.

### 3.3 Recommendations

The relatively high caregiver acceptability (81%) and preference demonstrated by the caregivers’ responses can be mainly attributed to the improved tolerability of the drug by the children, perhaps as function of the pellets’ improved palatability over the syrup formulation and the ability to easily co‐administer it with soft foods such as porridge. These findings are supported by various studies 9, 10, which report that these features of a medication are key factors which influence acceptability by caregivers. Although no direct comparisons were made between the pellets and syrup formulation in this study, the findings support the argument for solid formulations being more acceptable to caregivers of young children than what may be seen to be a more obvious solution of syrup formulations.

The study, however, demonstrated tolerability of the pellets to be an issue, with parents citing vomiting as a side effect in one in five children. This echoes findings from the CHAPAS2 study 8 which reported a higher rate of vomiting for children on oral pellets than the syrup formulation for children <1 year of age. While gastrointestinal upset is a common side effect with any formulation of LPV/r, this problem seems greater among young children. Ensuring correct administration of the pellets, which is more complex than that of the other formulations, becomes very important in this regard. When pellets dissolve in the food or liquid, this obviates the taste masking and reveals the unpleasant flavour of the medications, which may lead to the child vomiting or spitting out their food. Caregivers and healthcare workers in this pilot reported stirring the pellets in porridge as a common practice despite careful instruction being provided in the form of pictorial aides to avoid this practice. This is an important issue to address in the wider context of national rollout, as it can have negative implications on patient adherence 11 and overall uptake of the pellets.

In the light of the importance of correct administration and improving tolerability, one of the key recommendations from this pilot is that the messaging on how to administer the pellets should be supported by practical demonstrations from healthcare workers or expert mothers in peer support groups to ensure correct practice. Additionally, caregivers should be asked to demonstrate their understanding of the instructions provided before leaving the facility. Complementary to this education on pellet administration, caregivers should also be given pamphlets that highlight the potential side effects of the drug and ways to address them. This would encourage caregivers to better report adverse events and seek assistance on addressing challenges affecting their infant's tolerance of the medication.

Giralt *et al*. 12 are conducting the RE‐LIVING study, which aims at better understanding the factors that contribute to the acceptability and adherence to the new LPV/r oral pellets in three Kenyan settings. Final results are anticipated in 2019 and it is our hope to remain informed and guided by the evidence generated to further strengthen these recommendations on improving the administration and subsequent scale up of the pellets in Zimbabwe.

## 4 Limitations

This pilot originally expected to enrol 200 patients based on the availability of medications and paediatric patient loads at the selected sites; however, only 78.5% of the expected number was enrolled. According to health worker feedback, the main reasons for low enrolment included: caregiver reticence in signing the required consent forms (but not necessarily having resistance to initiation of pellets themselves), and initial discomfort on the part of the health workers with both instructing caregivers on the pilot procedures and with initiating paediatric patients. This feedback indicates that these issues may have related to research barriers rather than problems with the pellets, and thus, pilot enrolment may not be indicative of expected pellet uptake outside of the context of a pilot study.

Only 74 caregivers from the expected 157 responded to the survey. This pilot took place in public sector health facilities and questionnaires were administered by health workers in the facility who received no additional incentives from the study. While this realistic context provides insight into how pellets would be rolled out at scale, it also presented some challenges, which made it difficult to collect questionnaires from all the patients within the study time frames. In particular, patients returned for ART consultations on irregular schedules and health workers sometimes failed to complete the questionnaire.

## 5 Conclusion

This pilot supports that LPV/r oral pellets are a viable alternative formulation that patients and caregivers prefer for treatment of young children living with HIV. The results of this pilot support the substitution of pellets for other LPV/r formulation for children on ART in Zimbabwe's context and will inform the full scale rollout of this formulation nationally. The administration challenges noted should serve to guide further improvements in paediatric formulations beyond the oral pellets and contribute towards closing the gap between the optimal regimen options available to children and adults.

## Competing interests

The authors declare that they have no competing interests.

## Authors’ contributions

All co‐authors contributed in designing the operational study. BP and RK oversaw the data collection performed during the study. OS, through Drugs for Neglected Diseases Initiative (DNDi), supported the provision of LPV/oral pellets administered in the study. MLP and BP analysed the data, and worked with RK, TA and BS to write the first draft of the paper. All co‐authors contributed to interpreting findings and writing the manuscript.
